# Insight of Autophagy in Spontaneous Miscarriage

**DOI:** 10.7150/ijbs.68335

**Published:** 2022-01-01

**Authors:** Xue-Yun Qin, Hui-Hui Shen, Wen-Jie Zhou, Jie Mei, Han Lu, Xiao-Fang Tan, Rui Zhu, Wen-Hui Zhou, Da-Jin Li, Tao Zhang, Jiang-Feng Ye, Ming-Qing Li

**Affiliations:** 1 Laboratory for Reproductive Immunology, Hospital of Obstetrics and Gynecology, Shanghai Medical School, Fudan University, Shanghai 200080, People's Republic of China; 2 NHC Key Lab of Reproduction Regulation, Shanghai Institute for Biomedical and Pharmaceutical Technologies, Fudan University, Shanghai 201203, People's Republic of China; 3 Center of Reproductive Medicine of Ruijin Hospital, Shanghai Jiao Tong University School of Medicine, Shanghai 200025, People's Republic of China; 4 Reproductive Medicine Centre, Department of Obstetrics and Gynecology, Nanjing Drum Tower Hospital, The Affiliated Hospital of Nanjing University Medicine School, Nanjing, 210000, People's Republic of China; 5 Departments of Assisted Reproduction, Xin Hua Hospital Affiliated to Shanghai Jiao Tong University School of Medicine, Shanghai 200092, People's Republic of China; 6 Reproductive Medicine Centre, Affiliated Maternity and Child Health Care Hospital of Nantong University, Nantong, 226006, People's Republic of China; 7 Center for Human Reproduction and Genetics, Affiliated Suzhou Hospital of Nanjing Medical University, Suzhou Municipal Hospital, Gusu School, Nanjing Medical University, Suzhou 215002, People's Republic of China; 8 Medicine Centre for Human Reproduction, Beijing Chaoyang Hospital, Capital Medical University, Beijing, 100020, People's Republic of China; 9 Assisted Reproductive Technology Unit, Department of Obstetrics and Gynecology, Faculty of Medicine, Chinese University of Hong Kong, Hong Kong, People's Republic of China; 10 Division of Obstetrics and Gynecology, KK Women's and Children's Hospital, 229899, Singapore; 11 Shanghai Key Laboratory of Female Reproductive Endocrine Related Diseases, Shanghai, 200080, People's Republic of China

**Keywords:** autophagy, spontaneous miscarriage, trophoblast cells, placentation, decidualization, decidual immune cells

## Abstract

In some cases of spontaneous miscarriage (SM), the exact etiology cannot be determined. Autophagy, which is responsible for cellular survival under stress conditions, has also been implicated in many diseases. Recently, it is also surmised to be correlated with SM. However, the detailed mechanism remains elusive. In fact, there are several essential steps during pregnancy establishment and maintenance: trophoblasts invasion, placentation, decidualization, enrichment and infiltration of decidua immune cells (e.g., natural killer, macrophage and T cells). Accordingly, upstream molecules and downstream effects of autophagy are discussed in these processes, respectively. Of note, autophagy regulates the crosstalk between these cells at the maternal-fetal interface as well. Aberrant autophagy is found in villi, decidual stromal cells, peripheral blood mononuclear cells in SM patients, although the findings are inconsistent among different studies. Furthermore, potential treatments targeting autophagy are included, during which rapamycin and vitamin D are hot-spots in recent literatures. To conclude, a moderately activated autophagy is deeply involved in pregnancy, suggesting that autophagy should be a regulator and promising target for treating SM.

## Introduction

Spontaneous miscarriage (SM) refers to natural pregnancy loss before 20 weeks of gestation, though the exact definition remains controversial. It has drawn great social concern in light of increasing morbidity during latest decades 1. Main risk factors for SM include fetal chromosomal abnormality 2, maternal infections, uterine structural defects, endocrine disorders, and immune dysregulation 3, 4. However, in a certain part of cases, especially for recurrent spontaneous miscarriage (RSM), the underlying mechanisms still remain undefined, which urgently deserves more research on etiology, pathogenesis and effective therapeutic methods.

Macroautophagy/autophagy is a self-degradative cellular process in eukaryotic cells, warranting survival under stress circumstances like energy or nutrition shortage, inflammatory events, oxidative stress and endoplasmic reticulum stress 5, 6. Besides keeping cellular homeostasis, autophagy has been flagged the functionality in physiological and pathological process, for instance, immune response, malignancy, and neurodegeneration 7-9. Likewise, in reproductive system, autophagy has been reported to exist in physiological and pathological endometrium, involving in regulating menstrual cycle, development of endometriosis, adenomyosis and endometrial carcinoma 10-12. Additional studies have brought further supporting evidence to relationship between autophagy with embryogenesis, implantation, decidualization and pregnancy maintenance 13-15.

**Figure 1:**
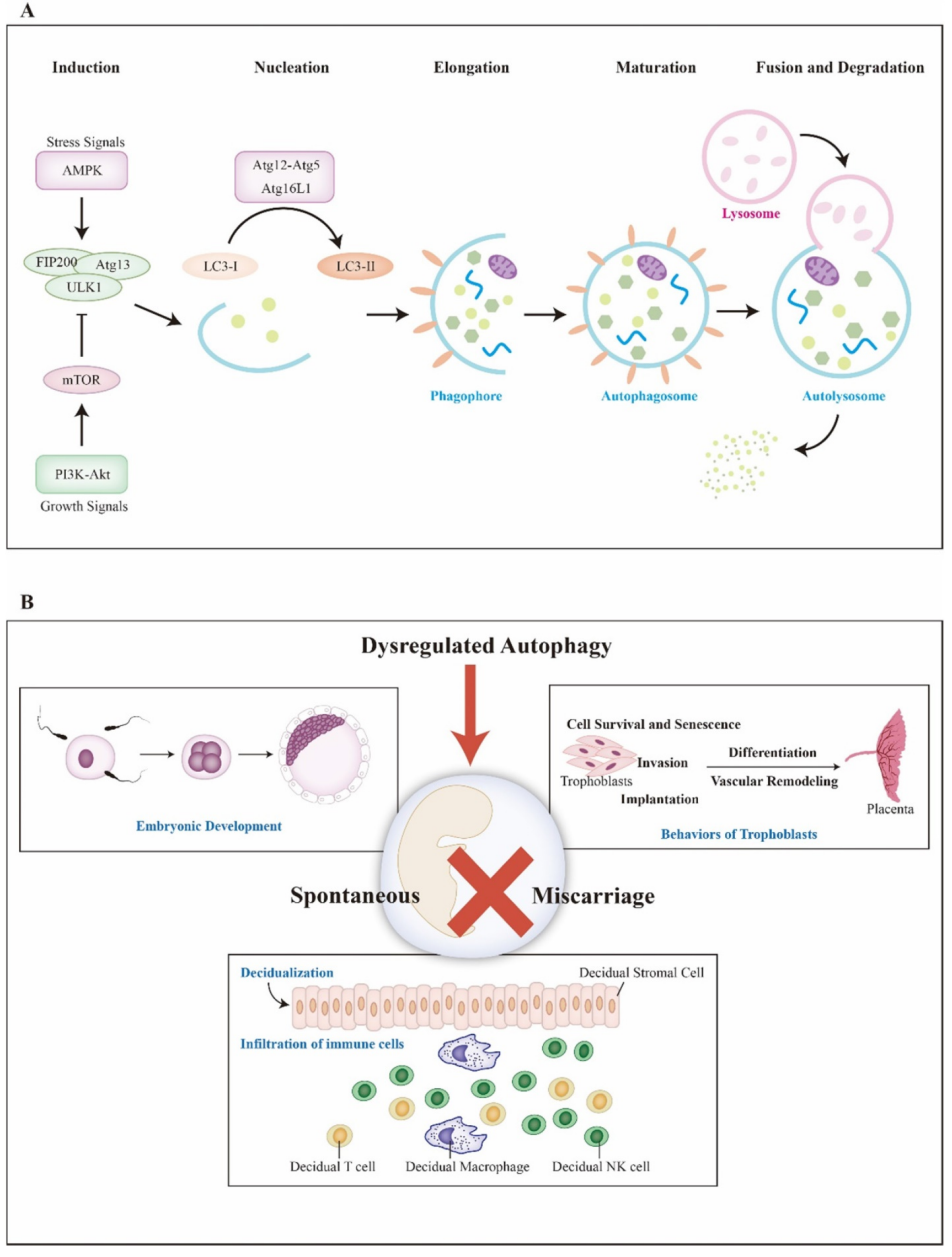
** Basic processes of autophagy and key events during pregnancy.** A) Induced by stress or growth signals, AMPK and PI3K-Akt are activated. Then, PI3K-Akt upregulates mTOR, an inhibitor of autophagy, to suppress ULK complex (ULK1-FIP200-Atg13). However, AMPK activates ULK complex to initiate autophagy. Autophagy can be divided into five basic steps, including nucleation, elongation, maturation, fusion and degradation. Atg16L complex (Atg5-12, Atg16L) help LC3 locate and insert into extending phagophore membrane. After that, mature autophagosome merges with lysosome and is degraded for cycling. B) Several key events during pregnancy are included. Aberrant autophagy might elicit miscarriage via affecting fertilization and embryonic development, bio-behaviors of trophoblasts, placentation, decidualization and infiltration of immune cells. **Abbreviations**: AMPK: 5' adenosine monophosphate-activated protein kinase; Atg: autophagy-related genes; LC3: microtubule-associated protein 1 light chain 3; mTOR: mammalian target of rapamycin; PI3K: class I phosphatidylinositol 3-kinase; ULK1: unc-51-like kinase 1

In mechanism, autophagy contains five basic steps: nucleation, elongation, maturation, fusion and degradation **(Figure 1A)**. First, autophagy is initiated by isolation membrane namely phagophore which later expands to engulf intracellular cargo and forms autophagosome. After that, autophagosome becomes mature via fusion with lysosome and the cargo is degraded for further recycle finally. In the aspect of molecule, autophagy is activated by the completion of unc-51-like kinase 1 (ULK1) complex, which consists of focal adhesion kinase family interacting protein of 200 kDa (FIP200), Atg13 and ULK1. ULK1 complex is negatively regulated by mammalian target of rapamycin complex 1 (mTORC1) and positively regulated by 5' adenosine monophosphate-activated protein kinase (AMPK) via directly suppressing and enhancing the activation of ULK, respectively 16. Followed with phagophore formation, Atg5-Atg12 conjugation interacts with Atg16L to form Atg16L complex. Meanwhile, LC3 precursor convert to LC3-II with the assistance of Atg4B and phosphatidylethanolamine successively 17. LC3-II locates and inserts into the extending phagophore membrane, typically serving as a critical marker of autophagy activity. Moreover, differing from non-selective autophagy, selective autophagy is regulated by the p62/SQSTM1 paradigm 18. Considering autophagy is a continuous cellular process, for proper detection, the concept of autophagic flux must be mentioned. To date, both bafilomycin A1, which inhibits lysosomal degradation and chloroquine, which blocks autophagosome-lysosome fusion, are used to evaluate the functional status of autophagic flux 19.

There are several significant events to maintain successful pregnancy: fertilization and embryonic development, trophoblasts invasion, placental development, decidualization, enrichment and infiltration of decidual immune cells **(Figure 1B)**. Recent studies imply that autophagy may associated with SM through regulating decidualization, trophoblast cells and immune cells at maternal-fetal interface 13, 20, 21. Herein, we discuss the importance of autophagy during early pregnancy and offer a new insight into spontaneous miscarriage and relevant treatment.

## Autophagy in fertilization and embryonic development

Fertilization is the beginning of embryonic development. For mammals, sperm cell penetrates in and donates its DNA to oocyte to form a zygote which will develop into a fetus later. Tentative evidence indicates autophagy might regulate both androcyte and oocyte during fertilization process 22-25. However, whether and how autophagy affects embryo development via regulating gametes remains unclear. In mouse embryos, autophagy is initiated from fertilization and shortly upregulated during preimplantation period. Zygote derived from knockout Atg5 oocyte and sperm fails to develop beyond the four- and eight-cell stages. But if adopts knockout Atg5 oocyte and wild type sperm, embryonic could continue to develop, indicating maternal Atg5 remained in oocyte cytoplasm might be a protective mechanism 26. Actually, defective embryo development one of the most common causes for SM 27. It has now been reported that autophagy trigged by virus disturbs embryo development during early pregnancy 28, 29. Besides, obesity could elicit embryonic DNA damage, aberrant histone methylation and impaired autophagy in mice 30. Survivin, a member of inhibitor of apoptosis protein family which regulates apoptosis and cell cycle, is involved in early embryo development via regulating spindle organization and chromosome alignment. Inhibited survivin leads to accumulated ROS and DNA damage, which induces autophagy and apoptosis in mice embryos 31. In RSM, an important ROS scavenger, preimplantation factor, is remarkably reduced. Subsequently, accumulated ROS might cause embryo toxicity via autophagy by modulating AMPK/mTOR 32, 33. These reports indicate that dysregulated autophagy induced by ROS and nuclear stress might impair embryo development, which further triggers pregnancy losses.

## Autophagy relates to trophoblasts and placental development

### Autophagy-related genes/molecules in trophoblasts

Trophoblast cells undertake the tasks of establishing the maternal-fetal interface and can be identified as villus trophoblasts (VTs) and extra-villus trophoblasts (EVTs). VTs are consist of inner layer of cytotrophoblasts (CTBs) and outer layer of syncytiotrophoblasts (STBs), which forms villus structure and enables maternal-fetal energy and nutrients exchange. EVTs mediate invasion, angiogenesis and vascular remodeling to facilitate placentation.

In normal pregnancy, LC3, a widely used distinguished marker for autophagy, is found to localize in VTs, EVTs and trophoblast-anchoring in term human placentae 34, 35. Confirmed by electron microscopy, the activity of autophagy predominantly localizes to the STBs with obvious autophagosomes. Meanwhile, other autophagic molecules including Beclin-1, Atg5, Atg9, Atg16L1, p62 and LAMP-2, are all exhibited in VTs 36. Some upstream regulators of autophagy have been identified to express in human trophoblasts as well. For instance, mTOR mainly expresses in STBs of human placenta and mediates amino acid transport. Fetal growth restriction (FGR) also exhibits mTOR inhibition and increased expression of its inhibitor tuberous sclerosis complex 2 37, 38. Besides, p53 is located in the first trimester placental CTBs and combats with LC3B-II levels in STBs. Similar results are also observed in BeWo cell line and placental explants 39. Bcl-2 is another suppressor of autophagy for its efficacy in binding with Beclin-1. In villus explants, Bcl-2 and its family member Bcl-xL are detectable in CTBs but negatively associated with expression of LC3-II 40. Transcription factor EB (TFEB), a positive regulator for autophagy and lysosomal genes, has been found to express in primary human trophoblasts and EVTs cell lines 41. However, for specimens taken from placentae of preterm birth patients, autophagy activity is reduced and related markers are differentially distributed. The expression of LC3 and Atg16L1, but not Atg7 or Beclin-1, are higher in STBs than those of CTBs 42. Compared with health controls, LC3 staining is elevated in STBs of SM patients 35. These studies display the expression patterns of autophagy-related markers in trophoblasts during normal human pregnancy and their aberrant alterations in pregnancy complications.

Likewise, autophagy-related markers are also exhibited in trophoblast cells of mice. LC3A, B and C mainly concentrate on STBs while giant trophoblast cells express LC3C instead of LC3A 43. The LC3B staining exists mostly in spongiotrophoblast cells with occasional expression in giant trophoblast cells 44. Induced autophagy could upregulate LC3B-II and p62 protein in mice placental trophoblasts 45. Trophoblast-specific conditional Atg7 knockout mice exhibits aberrantly accumulated proteins in placenta probably via reducing TFEB 41, implying an impaired autophagy plays important roles in trophoblast-associated pathological pregnancy conditions, such as preeclampsia (PE). Murine placental Atg4C and Atg7 declines in inflammation-induced preterm labor (IPTL) 46. IPTL has also been observed in Atg16L1-deficient mice with reduced ability to withstand infection 42. Overall, these results offer practical clues to autophagy in trophoblast cells. Expression patterns of autophagy-related molecules display in **Table 1**.

### Upstream regulators of autophagy for trophoblast cells

AMPK/mTOR has been identified as upstream regulatory pathway for autophagy. For one thing, AMPK, acting as a cellular energy sensor, is upregulated by energy consumption (elevated ratio of AMP to ATP) 47. Then, activated AMPK directly phosphorylates ULK 16. For another, inhibited class I phosphatidylinositol 3-kinase (PI3K) inactivates its downstream Akt and thus represses activity of mTORC1. After that, ULK complex frees from mTORC1 and drifts to an active form 48. Anyway, activated Atg13 and ULK, aggregates with FIP200 and forms ULK complex in order to initiate autophagy. However, either mTOR or AMPK seems to take an opposite effect on autophagy in trophoblasts. Zhang *et al.* have reported that moderate inhibition of mTOR enhances autophagy while excessive inhibition of mTOR reduces autophagy in trophoblasts. That could be mediated by the functional loop between the O-GlcNAcylation and phosphorylation of Beclin-1 49. Additionally, Yang *et al.* have reported AMPK as a negative regulator of autophagy in primary human trophoblasts 50. With the treatment of AMPK agonist, fusion and degradation of autolysosomes are impaired. In contrast, administration of AMPK antagonist upregulates LC3B-II and LC3B-II/I ratio with depletion of p62. Simultaneously, impeded mitophagy (decreased PINK1) and mitochondrion fission/fusion (decreased OPA1, MFN1, Drp1, and FIS1) are observed in cocultivation with AMPK antagonist or agonist 50. On top of different cell lines, whether AMPK antagonist could activate autophagy in an AMPK-independent manner deserves further research.

Inhibited Akt/mTOR promotes autophagy of trophoblast cells. LncRNAH19 targets miR-18a-5p, and the downregulation of H19 promotes autophagy via suppressing PI3K/Akt/mTOR *in vitro* 51. Insulin-like growth factor 2 (IGF2) inhibits autophagy as it's an inducer of PI3K/Akt 52. This effect could be blocked by miR-16-5p but be rescued by ligustrazine 53. Furthermore, as a downstream pathway of PI3K/Akt, Sonic Hedgehog signaling (Shh) could be inactivated by Akt 54, and thus enhances autophagy with accumulation of autolysosomes 20. Therefore, Akt/mTOR and Akt/Shh show inhibitory and promotive effects on autophagy respectively, which forms a functional loop.

Status of autophagy changes in response to various outer stress. Since hypoxia regulates CTBs proliferation and invasion to establish maternal-fetal interface during early pregnancy 55, the role of autophagy in this condition should be emphasized. In hypoxia, TFEB is downregulated and thus autophagic flux is disrupted in primary human trophoblasts 41. Reactive oxygen species (ROS) and endoplasmic reticulum (ER) stress also activate autophagy 56, 57 and upregulate LAMP-2 and LC3B-II, which could be confirmed by electron microscopy 36, 58. However, discrepancy does exist in status of trophoblastic autophagy under hypoxia 59, which might be explained by different samples, cell lines, severity of hypoxia and interpretation of trophoblast autophagy.

Actually, hypoxia-inducible factor-1α (HIF-1α), a transcription factor that stabilizes in hypoxia, is a critical upstream regulator for autophagy in trophoblasts. Elevated autophagy may act as a compensation for upregulated HIF-1α and aberrant morphological alterations of villus 60. In physiological hypoxia (8% O_2_), autophagy is activated by increased HIF-1α. Whereas, in severe/pathological hypoxia (∼2% O_2_), persistent overexpression of HIF-1α may impair autophagy in EVTs via soluble endoglin secreted by villus 61. Besides, hypoxia induces mitochondrial malfunctions and impaired mitophagy of trophoblasts 59, 62. BNIP3, a mitochondrial protein, competes with Beclin-1 for binding to Bcl-2. Thereby, extricated Beclin-1 triggers autophagy 63, 64. BOK and MCL1, both of which are Bcl-2 family members, tightly work as a system to regulate autophagy of trophoblasts in hypoxia. Tilted balance towards BOK induces autophagy which could be rescued by MCL1 65. These results suggest that Bcl-2 family members might mediate altered autophagy and dysfunctional mitochondria in hypoxia.

**Figure 2:**
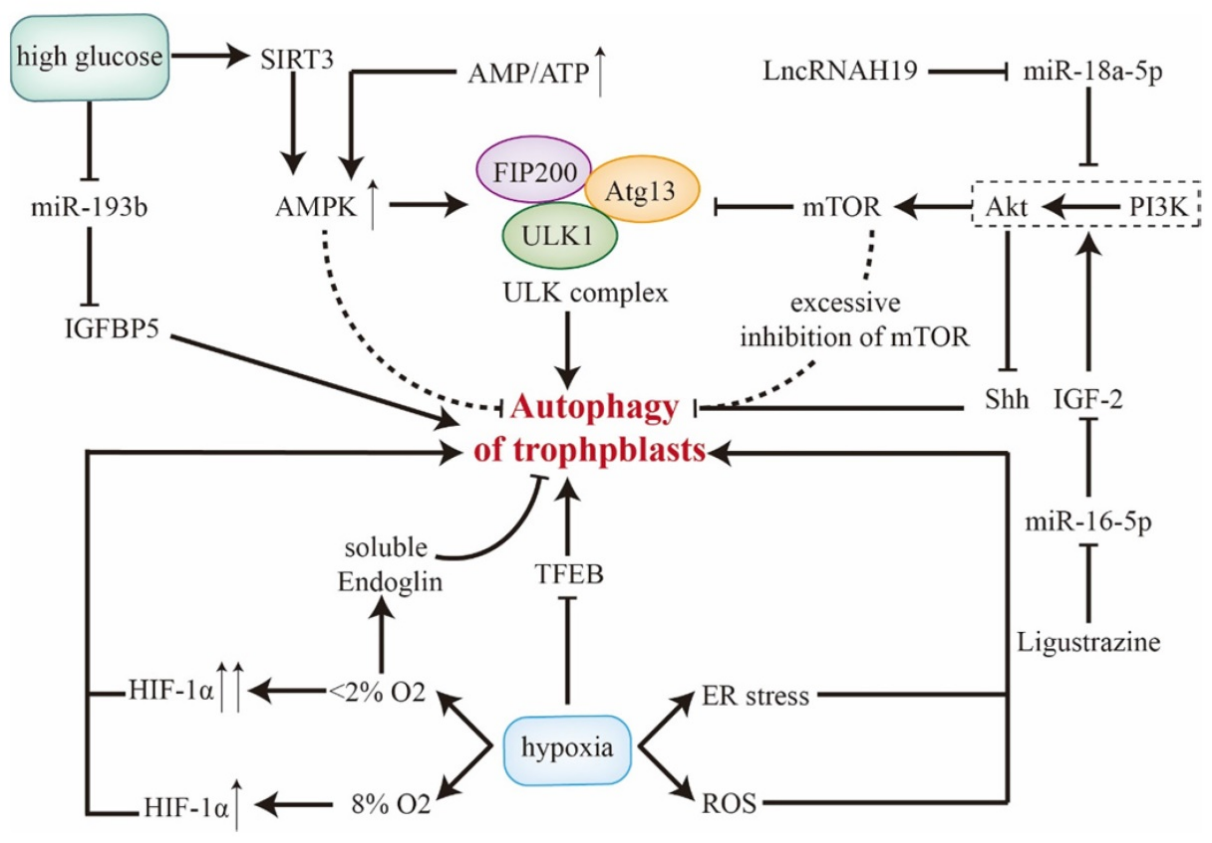
** Upstream regulators of autophagy in trophoblast cells.** The role of AMPK and PI3K/Akt/mTOR along with related molecules in regulating trophoblastic autophagy are included, during which AMPK and mTOR exhibit dual effects. Besides, SIRT3/AMPK and miR-193b in trophoblasts response for high glucose condition. Of note, different severities of hypoxia modulate autophagy discrepantly. **Abbreviations**: AMPK: 5' adenosine monophosphate-activated protein kinase; Atg: autophagy-related genes; ATP: adenosine triphosphate; ER stress: endoplasmic reticulum stress; HIF-1α: hypoxia-inducible factor-1α; IGFBP: insulin-like growth factor-binding protein; IGF2: Insulin-like growth factor 2; mTOR: mammalian target of rapamycin; PI3K: class I phosphatidylinositol 3-kinase; ROS: reactive oxygen species; Shh: Sonic Hedgehog signaling; TFEB: transcription factor EB; ULK1: unc-51-like kinase 1.

In high glucose condition, SIRT3/AMPK/mTOR activates autophagy and thus inhibits invasion and proliferation of trophoblasts, which could be abrogated through knockdown of Atg5 66, 67. Death-associated protein kinase-3 is elevated in placentae of gestational diabetes mellitus, facilitating autophagy flux and autophagosome-lysosome fusion by interacting with synaptosomal-associated protein 29 68. With treatment of high glucose, miR-193b is downregulated and elicits insulin-like growth factor-binding protein 5-induced autophagy, which may trigger gestational diabetes mellitus 69. However, in oxygen and glucose deprivation test, induced placental ER stress inhibits mTOR 38. Thereby, autophagy is remarkably enhanced with higher level of LC3B-II in primary CTBs, which could be reversed by re-supplementation 58. These results reveal that trophoblastic autophagy is initiated in both excessive and limited glucose conditions. Upstream regulatory signals of autophagy in trophoblast cells are displayed in** Figure 2**.

### Role of autophagy in regulating biologic behaviors of trophoblast cells

Trophoblast cell is crucial for embryo implantation and placental development, as well as of great significance for successful pregnancy maintenance, undoubtedly. And autophagy could regulate trophoblast behaviors via different gene expressions or signaling pathways.

#### Cell survival and senescence

Autophagy combats with hypoxia-induced apoptosis to facilitate placental trophoblast survival, in which Atg7 is involved in 70, while LC3 and Beclin-1 response differently to sever hypoxia (<1% O_2_) 71. Furthermore, activated autophagy restricts cell apoptosis via degrading caspase-9 in a p62-dependent manner 45. Sagrillo *et al.* have demonstrated that melatonin protects trophoblast cells from hypoxia via autophagy and Nrf2 pathway 72. Excessive ROS stimulates NLRP1 inflammasomes and promotes the secretion of IL-1β and CASP1. Meanwhile, both autophagy and apoptosis are increased. When facing ROS, silencing NLRP1 leads to decreased IL-1β, CASP1, and NLRP3. Then autophagy will take over to facilitate cell survival via decreasing p62 but elevating LC3-II, Beclin-1, Atg5, and Atg7. On the contrary, inhibited autophagy will elicit the expression of inflammasome 73. Therefore, hypoxia-induced ROS activates autophagy to ensure trophoblast survival. However, Lee *et al.* have reported autophagy as an inhibitor of trophoblast viability with accumulation of ROS and ER stress 74. Cha *et al.* also points out that TNF-α-induced autophagy mediates intrinsic apoptosis via Atg5 75, which could be rescued by resveratrol 76. Though different stress and cell lines they used may account for this discrimination, the possibility that autophagy regulates both adaptive response and damaging process in trophoblasts survival couldn't be ignored. Additionally, O_2_ concentration may play a predominant role in cellular senescence via autophagy in trophoblast cells. Physiological normoxia (5% O_2_) triggers autophagy, resulting in a decrease in senescence phenotypes (decreased SASP, IL-8, IL-6 secretion). While pathophysiological hypoxia (1% O_2_) causes cytotoxicity with the extracellular release of ATP and lactate dehydrogenase. Interestingly, administration of 3-methyladenine (3-MA), an autophagy inhibitor that blocks PI3K, reverses cellular senescence when facing oxygen stress (21% O_2_) 77.

#### Invasion

For rodents, conditional knockout of Atg7 in placental trophoblasts shows impaired invasion ability with reduced matrix metalloproteinases (MMP) 2 and 9 78. However, in human trophoblast cell lines, some studies have regarded autophagy as a negative regulator for trophoblast invasion 20, 51, 53, 79, 80. According to Zhou *et al.*, aberrantly enhanced autophagy might give rise to inhibited trophoblast invasion in SM patients 81. Downregulated Beclin-1 partially alleviates invasion inhibition caused by activated autophagy in JAR cell line 20. In mechanism, autophagy inhibition promotes trophoblast invasion via activating NF-κB pathway, upregulating MMP-2 and MMP-9 while downregulating TNF-α 80. TNF-α could also be induced by inflammatory stress, during which activated autophagy might be a protector for trophoblasts via suppressing NF-κB at a cost of suppressed invasion ability 82, 83. Besides, Cha *et al.* pointed out that autophagy induced by TNF-α mediates intrinsic apoptosis via Atg5, leading to cell death 75. Hence, autophagy regulates the functional loop between TNF-α and NF-κB in order to regulate trophoblastic invasion and apoptosis via MMP-2 and MMP-9. The exact role of autophagy in determining the fate and invasion ability of trophoblasts is inconsistent between these reports 80, 82, 83, which demands for further confirmations.

Mitochondrion is of great significance in cell motion and has been recognized as a regulator in migration/invasion of tumor cells and trophoblasts 84, 85. Invasion-defective human EVTs present disordered mitochondrial function with enhanced fission, decreased respiratory and membrane potential 84. Recent studies have worked on the role of autophagy in trophoblast invasion from the perspective of mitochondria. In primary human trophoblasts, aberrant autophagy might lead to impaired mitophagy (decreased PINK1) and disordered mitochondrion fission/fusion (decreased OPA1, MFN1, Drp1, and FIS1) 50. Besides, autophagy regulates mitochondrial transmembrane potential, mitophagy and mitochondrial biogenesis through BNIP3 62, 86, which is an autophagy inducer. SIRT1/AMPK/PGC-1α has been identified as an axis for governing mitochondrial biogenesis. In mouse myoblast cell line, human placental hydrolysate could rescue mitochondrial damage via autophagy through manipulating SIRT1/AMPK/PGC-1α 87. Whether this result applies to trophoblasts deserves more research. Anyway, impaired autophagy might lead to dysfunctional mitochondria. Interestingly, co-culture with human placenta-derived mesenchymal stem cells increases mitophagy (upregulated PINK1, ARKIN) in trophoblast cells and enhances invasion ability with increased MMP-2 and MMP-9 88. In all, there could be association between mitochondrial homeostasis and invasion ability of trophoblasts via mediating autophagy.

HIF-1α is another pivotal factor that mediates trophoblasts invasion via autophagy 89. Moderately upregulated HIF-1α serves as an energy source for EVT invasion via activating autophagy. Whereas, persistent overexpression of HIF-1α may impair autophagy in EVTs, which could be rescued by supplementation of ATP 61. Besides, the expression of HIF-1α is directly regulated in a NF-κB-dependent manner 90, rendering NF-κB-HIF-1α-MMP-2/9 as an important axis for autophagy to regulate trophoblastic invasion.

#### Implantation

Autophagy has also been demonstrated to participate in implantation 91, during which PI3K/Akt/mTOR is increased in luminal epithelium, decidual cells, embryoblast and trophoblast cells 92. In mice, mTOR reaches the peak during the period of 'implantation window'. By contrast, administration of rapamycin, a mTOR blocker, could reduce implantation sites, highlighting the necessity of mTOR for implantation 93. Compared with inter-implantation sites, Atg5, Atg12, LC3, cathepsin B, p62 and autophagosomes are significantly decreased at implantation sites 94. Studies above show inhibited autophagy during implantation, however there are some challenging results. Knockout of FIP200 in mice reproductive tract leads to implantation failures. FIP200 reverses the proliferation-blockage effect mediated by progesterone and thus facilitates endometrial receptivity 13. Particularly, autophagy might favor survival of blastocysts in delayed implantation model in mice 95.

### Autophagy regulates placentation

**Figure 3:**
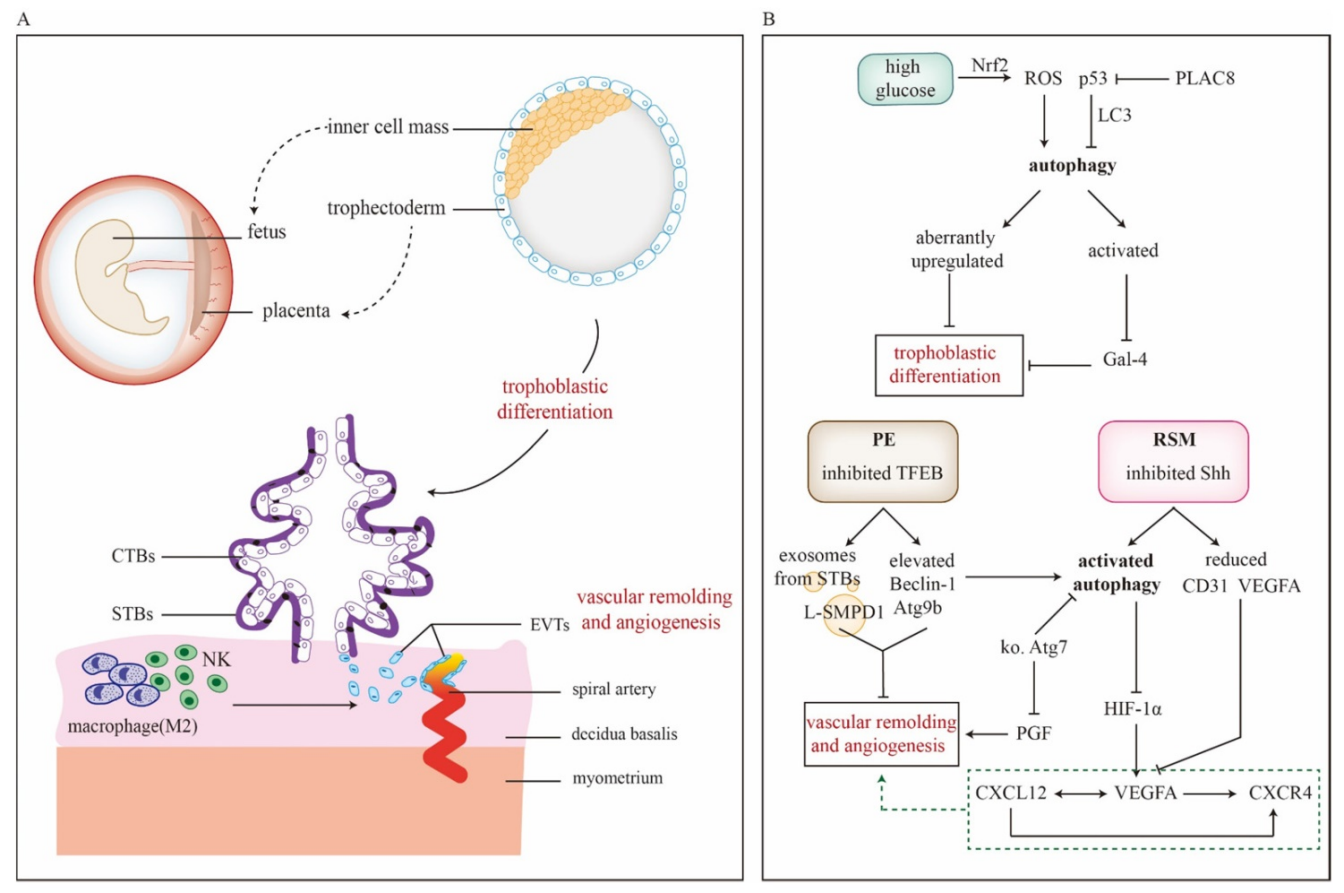
** Roles of autophagy in trophoblastic differentiation, placental vascular remodeling and angiogenesis.** A) Trophectoderm of blastocysts will further develop into placentae. In this process, trophoblasts go through differentiation into VTs and EVTs, which response for villus formation and vascular remodeling/angiogenesis, respectively. Additionally, decidual macrophages and NK cells can also facilitate placental vascular remodeling. B) The detailed mechanisms how autophagy regulates trophoblastic differentiation, placental vascular remodeling and angiogenesis are focused both in stress and pathological conditions (PE and RSM). In PE pathogenesis, TFEB is inhibited and thus upregulates Beclin-1 and Atg9b as well as promotes the exosomes containing L-SMPD1 releasing from STBs. By which way, vascular remodeling is impaired. Shh pathway, downstream of Akt, is suppressed in RSM. Moreover, it presents with activated autophagy and reduced VEGFA. Importantly, the CXCL12-VEGFA-CXCR4 axis is involved in angiogenesis, during which VEGFA relates to autophagy via the regulation by HIF-1α. **Abbreviations**: CTBs: cytotrophoblasts; CXCL12: C-X-C motif chemokine ligand 12; CXCR4: C-X-C motif chemokine receptor 4; EVTs: extra-villus trophoblasts; Gal-4: Galectin-4; HIF-1α: hypoxia-inducible factor-1α; ko Atg7: knockout Atg7; L-SMPD1: lysosomal sphingomyelin phosphodiesterase; NK cell: decidual natural killer cell; PE: preeclampsia; PGF: placental growth factor; PLAC8: Placenta-specific protein 8; ROS: reactive oxygen species; RSM: recurrent spontaneous miscarriage; Shh: Sonic Hedgehog signaling; STBs: syncytiotrophoblasts; TFEB: transcription factor EB; VEGF: vascular endothelial growth factor; VEGFA: vascular endothelial growth factor A.

Placenta is composed of decidua basalis, chorion frondosum and amniotic membrane. And it helps maintain pregnancy via providing oxygen, nutrients and immune defense for fetus. Based on trophoblast and other cells (fibroblasts, immune and vascular cells *etc.*), placental development goes through several hallmark events: implantation, trophoblastic differentiation and migration/invasion, decidualization, angiogenesis and vascular remodeling 96. So far, it has been reported that autophagy might involve in placentation (**Figure 3A** and **3B**). Expression levels of Atg5, Atg7 and Atg16L1 persistently increase during mice placental development, while LC3-II is scarcely detectable in placentae 97. Actually, LC3 family members differentially express during murine placentation, among which LC3B is the most susceptible to nutrient deprivation 43. There are several gestational complications tightly related to impaired placentation like PE, FGR, and SM, for which autophagy could be a regulator in pathogenesis. For example, LC3B-II, LAMP-2 and Beclin-1 increases in FGR placenta but mTOR and p62 declines 36, 98. Moreover, enhanced autophagy could inhibit transplacental amino acid transport via decreasing mTOR activity 37, 99. Folate deficiency also activates autophagy and thus jeopardizes placentation presented with smaller placenta and disordered endocrine function. Fortunately, impaired trophoblastic invasion ability could be reversed by 3-MA treatment 100. Induced autophagy could inhibit miR-30a-5p that targets Beclin-1 and cause pregnancy losses. Meanwhile, morphological alterations of mice placentae include reduced villi, aberrant aggregation of trophoblast cells, atrophy of interstitial and impaired vascular structure 101. However, there are some conflicting results display a different relationship between autophagy and placentation 44, 102. For instance, Atg2B gene is hypermethylated and downregulated in placenta of small-for-gestational-age new-born 102. Additionally, nutrient deprivation disturbs placental vascular remodeling system via suppressing autophagy and ER stress 44. These studies imply that placentation may work in a compensatory way to warrant fetal growth and inhibited autophagy could be an adaptive response to insufficient nutrient supply.

From the beginning of implantation, the trophoblast stem starts to differentiate into outer layer of primitive STBs and inner layer of CTBs, during which autophagic flux is activated 97. When implantation is completed, trophoblast develops into VTs and EVTs, which further mediates placental development. Arikawa *et al.* have reported that autophagy facilitates trophoblast differentiation through downregulating Galectin-4 to establish maternal-fetal interface 103. Defects of placentation in high glucose may attribute to ROS-induced autophagy via activating Nrf2 pathway and aberrantly increased expression of differentiation-related gene expressions (HAND1, MASH2, PL1, MMP12 and IGF2) 104. During differentiation, overexpression of p53 downregulates the level of LC3B-II, indicating p53 as a negative regulator of autophagy in trophoblast 39. Interestingly, p53 also serves as a functional link in the crosstalk between autophagy and apoptosis, regulating turnover and homeostasis of trophoblast cells 105. Placenta-specific protein 8 (PLAC8) co-localizes with p53 and assists its degradation. Autophagy induced by PLAC8 facilitates the viability and proliferation of trophoblasts and this effect can be abrogated by autophagy inhibitor chloroquine. In contrast, re-expression of p53 could partly reverse the PLAC8-induced autophagy activity 106. These findings flag the significance of autophagy in trophoblast differentiation during placental development.

Spiral artery modifications are conducted by EVTs. Interstitial trophoblasts penetrate the decidua and adjacent myometrium, and their aggregation around the arteries makes preparation for invasion of endovascular trophoblasts to build low-resistance blood flow at maternal-fetal interface. During that time, the endovascular trophoblasts form a plug at the terminal of spiral arteries until full utero-placental circulation is established at the end of first trimester 107. The conditional knockout of mice placental Atg7 shows downregulated placental growth factor with inhibited invasion and vascular remodeling of EVTs 78, suggesting necessity of autophagy for physiological placentation. However, autophagy is also involved in other pathological conditions. In RSM, inhibited Shh pathway might enhance autophagy and reduce vascular endothelial growth factor A (VEGFA) and CD31 expression, leading to aberrant placental angiogenesis 20. TFEB is a master transcriptional regulator of lysosomal biogenesis and autophagy. Decreased TFEB inhibits lysosomal protein expression and activates mTORC1 in autophagy-defective EVTs 41. Of note, TFEB also elevates autophagy of PE development via exosomes. For one thing, Beclin-1 and Atg9b, both of which are downstream targets of TFEB, increase in the STBs. For another, exosomes containing active lysosomal sphingomyelin phosphodiesterase 1 (L-SMPD1) forms at the membrane of STBs and is released into maternal circulation. As a result, circulating L-SMPD1 might cause impaired placental angiogenesis 108. Autophagy induced by inhibited protein kinase C β represses VEGFA-mediated human umbilical vein endothelial cells tube formation 109. Studies above imply that autophagy dysregulates placental angiogenesis. Aberrant alterations in immune cells induce impaired placentation as well. Poor vascular remodeling of placentae could be observed in NK cell depletion mice 21. Nakabayashi *et al.* have clarified that populations of decidual immune cells in PE placenta, including decidual CD3^+^T cells, CD8^+^T cells, CD4^+^T cells, Foxp3^+^Treg cells, CD56^+^NK cells, and CD68^+^macrophages, are smaller than those of control. Meanwhile, p62 is significantly higher in EVTs of PE 110. Additionally, changes in mitochondria are associated with vascular remodeling during first trimester. Mitochondrial respiration decreases at 11 weeks and the content increases at 12-13 weeks, which parallels to onset of utero-placental circulation. Increased respiration might be an adaptive response to ischemia-reperfusion 111. In PE placentae, three major mitochondrial malfunctions are observed, including distorted mitochondrial fusion, disordered mitophagy and diminished mitochondrial biogenesis 62, 112. Anyway, these reports indicate the significant role of autophagy in angiogenesis and vascular remodeling during placentation.

Particularly, C-X-C motif chemokine ligand 12 (CXCL12) and C-X-C motif chemokine receptor 4 (CXCR4) signaling pathway might regulate angiogenesis via autophagy to ensure placental development. CXCL12 upregulates expression of VEGFA, which can further stimulate CXCL12 synthesis and increase secretion of CXCR4, a receptor for CXCL12. Hence, it's a positive feed-back loop with great potential to favor angiogenesis 113. CXCL12 inhibition leads to a decrease of VEGFA and HIF-1α. Meanwhile, autophagy in endometrium is increased via suppressing Akt/mTOR 114. Besides, HIF-1α could bind with VEGFA promoter to stimulate its expression 115. These results may unveil how CXCL12-CXCR4 axis regulate angiogenesis via modulating autophagy at maternal-fetal interface. However, CXCL12 inhibition increases CD34^+^ hematopoietic stem cells 114. Whether the greater recruitment is a compensation for aberrant angiogenesis needs more research.

## Autophagy and decidualization

### Level of autophagy during decidualization

Decidua is composed of decidual stromal cells (DSCs), glands, immune cells, blood and lymph vessels, among which DSCs take up a major part. Decidualization refers to a process that endometrium highly develops into secretory lining via continuous stimulation of estradiol (E2) and progesterone (P4), making preparations for the implantation of blastocyst 116. Subsequently, immune cells infiltrate and endometrial stromal cells (ESCs) differentiate into DSCs with accumulation of glycogen and lipid droplets. In this process, a series of proteins, cytokines and growth factors are secreted. For instance, insulin-like growth factor-binding protein 1 and prolactin, both of which are widely-used markers for well-established decidualization 117, positively associated with cyclic adenosine monophosphate (cAMP) which is an inducer of decidualization.

Regarding decidualization is energy-dependent for its frequent metabolism and biosynthesis, autophagy may server as an energy resource for metabolites recycling. According to Oestreich *et al.*, autophagy is negatively correlated with level of ATP during human decidualization. The positive regulators of autophagy, acetyl-CoA carboxylase, phosphorylated-ACC and p-ULK1 are increased in decidua 118. There also shows increased LC3-II and decreased p62 in decidua when compared with proliferative or secretory phases of endometrium tissues 94, 119. In contrast, administration of 3-MA, impedes decidualization of mice 94. Likewise, our previous research has also revealed upregulated LC3, LC3II/I and decreased p62 in DSCs. However, in SM patients, autophagy of DSCs is suppressed with poor dNK residence, which could be alleviated by rapamycin 21. After treatment of bafilomycin A1, LC3B-II tends to increase, suggesting enhanced autophagy flux 118. Similar result is also confirmed by chloroquine 120. Studies above indicate dynamic autophagy flux is activated during human ESC decidualization. **Table 1** shows the expression of autophagy-related markers on decidua or DSCs.

### Regulation of autophagy in decidual stromal cell

Transcription factors homeobox A10 (HOXA10) and forkhead box O1 (FOXO1) are positive regulators for decidualization 121. PI3K/Akt is activated during poor decidualization of ESCs derived from endometriosis, leading to decreased insulin-like growth factor-binding protein 1 via downregulating nuclear FOXO1 122. FOXO3, another member of FOXO family, shares the ability of inhibited by Akt 123. Activation of FOXO3 induces autophagy flux and its inhibition exerts opposite effect, which has been verified in kidney 124 and neural stem cells 125. Similarly, Akt/MMP and FOXO3a/autophagy flux are downregulated in defective DSCs when exposing to microgravity 126. HOXA10, a decidual marker, has been reported to interact with autophagy via cathepsin L which degrades autolysosomes. This interaction could be inhibited by folate-deficiency via dysregulating AMPK/mTOR pathway, thereby leading to impaired decidualization of mice 127. On top of inhibiting autophagy, folate deficiency represses apoptosis of DSCs through downregulating the ratio of Bax to Bcl-2 induced by ROS in early pregnancy 128. Anyway, folate deficiency impairs autophagy of DSCs via manipulating AMPK/mTOR, leading to defective decidualization.

Decidualization is primarily mediated by E2 and P4 through interacting with progesterone receptor 129. Oppositely, high insulin pregnant mice present defective decidualization and show reduction in serum E2, P4, FSH and LH levels 130. Interestingly, Shakeel *et al.* have proposed that progesterone-associated membrane component 1 could bind with LC3 and is necessary for degradative activity of autophagy in lung cancer and kidney cell lines 131. Actually, in endometrium, both autophagy and apoptosis increase significantly during secretory phase, peaking at late-secretory phase 132. We have also found that P4 induces autophagy of ESCs at secretory phase while E2 takes an inhibitory effect 133. These results imply P4 might be a key factor for upregulated autophagy during decidualization. However, it seems that P4 doesn't involve in impaired decidualization induced by high-fat/high-sugar diet. High-fat/high-sugar diet induces impaired autophagy and markers for well-established decidualization (insulin-like growth factor-binding protein 1 and prolactin) are lower expressed in primary human ESCs without alterations in serum level of P4 118. Additionally, treating DSCs with mycophenolic, an immunosuppressive drug, autophagic flux and senescence were dramatically activated in response to nuclear stress, presented with a reduction in pre-rRNA synthesis and disruption of the nucleolus. Subsequently, p53 is stabilized by nuclear stress and thus generates a p21-mediated cell cycle arrest in late S and G2 phases, inhibiting proliferation of DSCs 134. Considering we have discussed p53 as a negative regulator of autophagy before, it could be surmised that autophagy of DSCs during decidualization might be regulated in a nuclear stress/p53/autophagy flux loop to reduce the cellular damage caused by stress.

### Autophagy facilitates decidualization

As mention above, it's reasonable to deduce that autophagy probably facilitates decidualization during early pregnancy. In contrast, autophagy inhibition induced by 3-MA results in abnormal decidualization with smaller robust deciduomas, decreased uterine horn gross weight and unclear cellular boundaries 94. Remarkably, autophagy-related proteins work differentially in mechanism. As for FIP200 which makes up ULKI complex, it mediates P4-induced decidualization. Depletion of FIP200 impairs decidualization and endometrial receptivity both in mice and human ESCs 13. After initiation of autophagy, Atg12 is activated by Atg7 then is transferred to interact with Atg10, and forms Atg12-Atg5 complex finally. With conjunction of Atg16 or Atg16L1, an exquisite complex is established for elongation of phagophores 17, 135. Nevertheless, Atg5, Atg7 and Atg16L1 are not entirely similar in their role of regulating autophagy during decidualization. Interestingly, knocking out Atg5 has a stronger effect on blocking autophagy flux in ESCs than knocking out Atg7. But silencing Atg7 is more effective in impairing decidualization 120, suggesting Atg7 might facilitate decidualization in an autophagy-dependent manner. Moreover, expression level of Atg16L1 remains consistent during decidualization, while knocking out ATG16L inhibits LC3B-II level 119. Anyway, these results may indicate positive relationship between autophagy and decidualization. However, high insulin disturbs endometrial angiogenesis and decidualization via enhancing autophagy (increased Atg5, Beclin-1 and decreased p62) 130. Whether elevated autophagy could be a compensation for impaired decidualization in response to insulin and the underlying mechanism still needs more supports.

### Autophagy and decidual immune cells

#### Decidual NK cell

Natural killer (NK) cell, a cytotoxic lymphocyte, plays important roles in both innate and adaptive immune response, and induces apoptosis via releasing granzyme B and perforin or mediating Fas/FasL pattern. The phenotypes and activity of NK are modulated during its development and maturation, and CD3^-^CD56^+^ has been identified as the specific marker for NK in adults 136. Of note, expression levels of CD56 are closely related to NK cytotoxicity. CD56^dim^ NK takes a percentage over 90 in total NK population and mainly exists in peripheral blood with highly expressed CD16, which facilitates antibody-dependent cellular cytotoxicity. While CD56^bright^ CD16^-^ NK is dominant in secondary lymphoid tissues and endometrium, which secrets abundance of cytokines, for instance TNF-α, INF-γ, IL-12, IL-15 and IL-18, in order to regulate immune microenvironment 136, 137. Additionally, the activity of NK is determined by the functional balance between its inhibitory (KIR, NKG2, *etc.*) and stimulating (FCGR3, NCR, *etc.*) receptors 138. Dysregulation of phenotypes and cytotoxicity of NK could contribute to a series of diseases, including malignancy, infectious diseases, and endometriosis 139-141.

Decidual NK (dNK) cells are recruited in decidua, migrating through DSCs with the assistance of various chemokines like CXCL10 and CXCL12. Meanwhile, the expression profiles of chemokine receptors on dNK are altered in coculture with DSCs 142. Besides, dNK cells could differentiate from uterine hematopoietic progenitor cells as well as directly derive from CD16^+^ peripheral blood NK cells 143. Though there is a declining trend throughout gestation, dNK is dominant among decidual immune cells during early pregnancy 144. Autophagy could regulate the crosstalk between dNK and DSCs at maternal-fetal interface 145. Our recent research shows increased autophagy of DSCs promotes NK residence mediated by MITF-TNFRSF14/HVEM-MMP9-adhension molecules axis during decidualization. However, SM patients display insufficient dNK cell residence and impaired autophagy of DSCs. Despite dNK presents with higher level of autophagy than that of NK from peripheral blood, it doesn't regulate its own adhesion and residence directly. We also found NK depletion mice presented poor vascular remodeling 21. Actually, dNK is also capable to induce decidual angiogenesis owing to secretion of various isoforms of vascular endothelial growth factor 146, but whether it's autophagy-dependent remains unclear. In all, autophagy of DSCs could facilitate dNK residence in order to build fully functional decidua.

dNK enhances trophoblast invasion and promote vascular remodeling in early pregnancy 146, 147. Admittedly, autophagy mediates subtle connection between dNK and trophoblast cells. Autophagy-deficient trophoblasts increases the cytotoxicity of NK cells via upregulating IGF-2 and impedes trophoblasts invasion by downregulating paternally expressed gene 10 148. Population of dNK is smaller in PE placenta which might account for aberrant vascular remodeling of EVTs 110. Liu *et al.* have demonstrated that UL16-binding protein 1 interacts with NKG2D receptors on dNK, which downregulates the expression level of NKG2D and thus alters the balance of cytokine secretion with elevated TNF-α, IFN-γ, TGF-β1, IL6 and IL-8. Subsequently, this imbalance contributes to impaired invasion of EVTs 149. Recently, they have further reported that UL16-binding protein 1 might impair trophoblast invasion depending on TNF-α secreted by dNK, which activates autophagy via suppressing NF-κB 83.

Studies of autophagy and uterine NK (uNK) based on ESCs may offer us more ideas in exploring DSCs. The axis of autophagy/STAT3/HCK in ESCs has been reported to regulate the cytotoxicity of uNK. In detail, autophagy suppression reduces HCK via downregulating STAT3 and thus upregulates its downstream CXCL8/IL-8 and IL-23A, resulting in increased FCGR3^-^ NK cells. PTGS2, which is targeted by miR1185-1-3p, also upregulates FCGR3^-^ NK cells in endometriosis 150. Ectopic lesions of endometriosis secret abundant IL-15, which inhibits apoptosis but promotes invasion, viability and proliferation of ESCs in an autocrine manner. Meanwhile, excessive IL-15 blocks cytotoxicity of NK cells with decreased granzyme B, IFN-γ, activating receptor natural-killer group 2D (NKG2D) and natural cytotoxicity receptor NKp44. Rapamycin, an autophagy inducer, reduces the expression of IL-15 receptors on ESCs, such as IL-15Rα and IL-2Rβ, indicating its therapeutic potential in treating endometriosis 151. Protopanaxadiol could reverse the inhibitory effect mediated by E2 on autophagy of ectopic ESCs and induce NK cytotoxicity. The expression of NKG2A, activated natural cytotoxicity receptors and IFN-γ are dramatically elevated on NK with decreased IL-10 level 152. Whether these regulatory mechanisms still fit in dNK and DSCs needs further confirmation.

#### Decidual macrophage

Macrophage, mostly derived from peripheral monocytes, residents in various organs and tissues, including decidua. During early pregnancy, macrophage takes up the second largest population of decidua immune cells, only secondary to dNK 144. Autophagy is of great significance for the differentiation and survival of monocytes. Macrophage colony-stimulating factor (M-CSF) facilitates macrophages differentiating from monocytes and remarkably activates AMPK via ROS produced in a M-CSF receptor-dependent way. Then, enhanced AMPK induces autophagy flux in a FOXO3-dependent manner 153. Autophagy could also be induced via freeing Beclin-1 from Bcl-2 by activating JNK and blocking Atg5 cleavage, contributing to monocyte-macrophage differentiation 154. Moreover, M-CSF secreted by DSC induces CD14^bright^CD163^+^CD209^+^CD86^dim^ phenotype of decidual macrophage and promotes macrophage survival 155.

According to functional characteristics, macrophages could be divided into two main groups: M1 and M2. M1 macrophage, classically activated through lipopolysaccharides and IFN-γ, generates abundance of IL-12 to mediate inflammation. M2 macrophage, alternatively activated, secrets anti-inflammatory cytokines IL-10 156. IL-10 mediates the production of VEGF for tissue repairing and vascular remodeling under hypoxia 157. Co-culture with DSCs makes M1 shift to M2 phenotype^158^, and other studies have also verified decidual macrophage as M2 phenotype owning to its role in tissue repairing and remodeling 144, 159. The recruitment of M1 or M2 could be modulated by various chemokines and signaling pathways. For example, M1 is summoned by CCL19/CCL21 which could be blocked by PI3K 160, indicating the potential of autophagy in facilitating M2 polarization and residence in decidua. Of note, aberrant polarization of M1-M2 may account for several pregnancy complications, such as RSM, FGR and PE, for its role in implantation, decidualization and angiogenesis 161. HIF-1α directly binds to VEGF transcription promoter and upregulates VEGF which induces recruitment and polarization of decidual M2 under hypoxia 115, 158. Zhang *et al.* have elucidated that inhibited hypoxia-induced autophagy downregulates HIF-1α/STAT3 with decreased VEGF 162. In addition, autophagy might promote placental angiogenesis through VEGF via modulating CXCL12/CXCR4 as discussed before. These findings indicate that autophagy might positively regulate polarized M2 and be responsible for placental angiogenesis.

Decidual macrophage, mediating immunosuppressive effect at maternal-fetal interface, secrets IL-10 and expresses indoleamine 2,3-dioxygenase (IDO) to impede activation of T cells. Moreover, aberrant subgroups of decidual T present elevated regulatory T (Treg) cells and decreased naïve T cells 163. IL-10, an anti-inflammatory cytokine, is verified to reduce among RSM patients 164, with alteration in both its promoter and intron polymorphisms 165. As for murine macrophage, IL-10 inhibits the activation of autophagy via PI3K signaling pathway 166. In asthma, inhibiting autophagy of airway macrophages is entitled to induce immunosuppressive effect mediated by IL-10 167. These results indicate that IL-10 produced by decidual macrophage might decrease and thus impair immune tolerance in RSM patients. IDO, which is also expressed by EVTs, maintains immune tolerance by suppressing T-cell response 168. IDO expression could reduce embryo absorption rate via increasing Treg and alleviating inflammatory response in RSM models 169. However, there are not relevant articles directly about autophagy of decidual M2 macrophage in regulating maternal immune tolerance, and the relationship among IDO, autophagy and immune regulation seems complicated in other cell lines and samples. IDO-mediated immunosuppression activates mTOR signaling pathway to facilitate T-cell tolerance 170. Upregulated IDO induced by IFN-γ could dramatically enhance autophagy and activate phagocytosis of macrophage, inhibiting growth of cervical cancer cells 171. Whereas, Chinnadurai *et al.* have reported that IDO-induced immunosuppression works in an autophagy-independent manner 172. Taken together, decidual macrophages are involved in maintaining immune tolerance via upregulating Treg, whether autophagy influences this process and how IDO and IL-10 regulate immune milieu at maternal-fetal interface still remain elusive.

#### Decidual T cell

Decidual T cells regulate maternal-fetal immunity in an intricate way for their various functions and phenotypes. In early pregnancy, 30-45% of decidual T cells are CD4^+^ T cells and 45-75% are CD8^+^ T cells. CD4^+^ T cells could be decided into Th1, Th2, Treg and Th17 subgroups based on their transcription expression factors and secreted cytokines. They comprise 5-30%, 5%, 5%, 2% of total population of decidual CD4^+^ T cells respectively, and are differently regulated in order to maintain pregnancy 173. The axis of Th1/Th2 is regarded as a classical pattern that is involved in maternal-fetal immune tolerance. Th1 is dominant during pre-implantation period and favors trophoblast invasion, after that Th2 takes over to warrant optimal conditions for fetal development and placentation. Tilted balance towards Th1 contributes to pregnancy disorders, such as PE and RSM 174. Actually, cytokines generated by Th1 and Th2 could further influence the level of autophagy. Autophagy is enhanced by IFN-γ and TNF-α fromTh1, and is suppressed by IL-4, IL-13 and the anti-inflammatory cytokine IL-10 from Th2 175. Treg (CD4^+^CD25^+^Foxp3^+^) cells are recruited before implantation period and produce immunosuppressive cytokines like IL-9, IL-10 and TGF-β to help build immune tolerance. Besides, Treg cells can be induced by VEGF milieu of M2 through increased programmed death-ligand 1 (PD-L1) 176. Of note, PI3K/mTOR, an inhibitory pathway upstream of autophagy, has been reviewed as a regulator in development, function, and stability of Treg cells 177. Th17 is negatively associated with female fertility 178, and its related cytokines (IL-23, IL-17, IL-6) upregulate in SM 179. Whereas, no statistically significant polymorphic site of IL-17A/F is observed for susceptibility of SM 180. Particularly, autophagy has been reported to protect organ injuries caused by Th1 and Th17 in sepsis 181, indicating autophagy might balance Th1 and Th17. Anyway, both Th1/Th2/Th17 and Treg paradigms modulate immunological milieu in normal pregnancy and several pregnancy complications 182. Though the role of autophagy in decidual T cells during gestation has not been reported so far, we could still get some insights from context.

Maternal E2 and P4 exert pivotal modulatory properties on initiation and maintenance of conception, which also elicits polarization of CD4^+^ T cells. P4, which is involved in implantation and decidualization, upregulates Th2, Treg and downregulates Th1 and Th17. E2, which is involved in endometrium receptivity, promotes Treg and Th2 while suppresses Th1, probably through TGF‑β-mediated IDO expression 178, 183. Besides, it has been well described that E2 and P4 regulate autophagy through interacting with estrogen receptors (ER) and progesterone receptors (PGR), respectively 10, 184, 185. In fact, ER and PGR have been verified to express on T lymphocytes in human, and downregulation of their receptors is tightly correlated with SM 186. However, there are few reports about effects of E2 and P4 on autophagy of T lymphocytes yet. Taken together, ovarian hormones might regulate polarization of CD4^+^ T cells mediated by autophagy via ER and PGR, which still remains to be confirmed by further studies.

Transforming growth factor beta (TGF-β) regulates proliferation and differentiation of T cells 187 and corporates with IL-10 to suppress Th cells 182. TGF-β/SMAD signaling is associated with pregnancy and fertility for its role in endometrium receptivity, implantation 188, proliferation and invasion of trophoblasts 189 and decidualization 190. Notably, emerging research suggest TGF-β could induce autophagy with increased Beclin-1, Atg5 and Atg7 in other cell lines 191, 192. Thereby, autophagy is supposed to modulate decidual T cells by means of TGF-β during pregnancy. Chronic endometritis patients usually suffer from recurrent implantation failures and their serum present higher levels of IL-17 but lower levels of IL-10 and TGF-β in endometrium. Simultaneously, autophagy locates in ESCs where LC3 increases with inhibited mTORC1 193. That indicates autophagy in ESCs may disturb the endometrial receptivity, immune milieu and implantation via mediating shift from Treg towards Th17. Soluble endoglin produced by trophoblasts not only inhibits autophagy but also dysregulates the differentiation of Treg cells by downregulating TGF-β, participating in the development of PE with invasion failure of trophoblasts 194. Moreover, TFEB, an inducer of autophagy, is critical for TGF-β-induced migration and metastasis of pancreatic cancer cells 195. TFBE also promotes releasing of ICAM-1, a molecule for T cell adhesion, to impair placental angiogenesis via exosomes 108. Whether autophagy regulates decidual T cell adhesion via TGF-β and how it modulates crosstalk among decidual T cells, trophoblasts and ESCs/DSCs should be an interesting topic to explore.

## Autophagy disorder and spontaneous miscarriage

### Aberrant autophagy in SM patients

Around 15% pregnancies end up with SM, which is characterized by pregnancy loss before the 20^th^ week. In particular, RSM refers to occurrence of 3 or more consecutive miscarriages, and over half of cases show no identifiable causes 196. Accumulating evidence indicates the important role of autophagy because of aberrant expression of autophagy-related markers in SM/RSM (**Table 2**). LC3, LC3II/I, Atg5 and Beclin-1 are higher in RSM placentae 81, 197 along with inhibited Shh signaling pathways 20. Avagliano *et al.* have also shown that STBs in SM exhibit a higher density of LC3 staining with more autophagosomes than those of normal controls. HIF-1α increases in SM villi and decidua, while the ratio of Bax to Bcl-2 and cleaved caspase 3 are elevated in decidua of SM, without statistical significance, though. Thus, autophagy might be a protector from hypoxia-induced apoptosis 35. Interestingly, as compared to women with their first conceptions, autophagy of peripheral blood mononuclear cells increases in women with prior spontaneous pregnancy losses but no deliveries 198. However, here exists some discrepancies. Yang *et al.* have shown that plasmacytoma variant translocation 1 (PVT1) is decreased in villi from RSM patients. In vitro, PVT1 knockdown elicits impaired autophagy with increased mTOR, p62 and decreased Beclin-1, LC3- II/I, ULK1 in HTR-8/SVneo cell line 199. Our previous studies display that autophagy is impaired in RSM. Smaller number of autophagosomes with aberrant distribution are exhibited in the villi of RSM patients 148. Insufficient autophagy with lower Atg5, LC3B and higher p62 is observed in DSCs derived from SM patients. Besides, downstream molecules of autophagy mediating NK residence are also reduced 21. Wei *et al.* have confirmed these results in rat model induced by antiphospholipid antibodies. Elevated p62 and mTOR as well as downregulated Beclin-1 and LC3-II are presented in placentae and the impaired autophagy could be rescued by rapamycin 200. Except for different ways to evaluate of autophagy, cell types and sampling, various pathogenic mechanisms involved in could still be to blame for the inconsistence. Of note, autophagy is a dynamic process and might act as a compensation for cellular stress in SM. Furthermore, the exact causal relationship between altered autophagy and SM remains to be established based on larger scale of subjects and well-designed studies.

### Potential therapeutic methods targeting autophagy for SM

In normal pregnancy, Yin Yang 1 (YY1) can interact with plasmacytoma variant translocation 1 (PVT1) promoter to upregulate its transcription, which further favors trophoblasts invasion and adhesion by autophagy via mTOR signaling. Effects followed with decreased PVT1 in villi samples might be involved in RSM pathogenesis, rendering PVT1 as a therapeutic target 199. PLAC8, a newly-explored factor in tumorigenesis, has been regarded as a target of treating oral squamous cell carcinoma for its regulatory roles in ERK^201^, Wnt/β-catenin and PI3K/Akt/GSK3β pathways 202, respectively. In trophoblasts, PLAC8 co-localizes with p53 and mediates its degradation, facilitating the viability, proliferation and differentiation via enhanced autophagy 106. Punicalagin enhances autophagic flux to protect primary human STBs from apoptosis induced by stress conditions 203. Hyperoside activates autophagy and combats inflammatory response in rat model with decreased IL-1β, IL-8, ICAM-1, VCAM-1 and complement C3, thereby rescues pregnancy losses via downregulating mTOR/S6K and TLR4/MyD88/NF-kB 200. Studies above offer us insights into treatment of RM targeting autophagy. Rapamycin, a mTOR inhibitor to induce autophagy, could also reverse adverse pregnancy outcomes and elevates LC3 stanning in junctional and labyrinth zones in placentae of rats 200. It effectively induces autophagy to reduce NK cytotoxicity in trophoblasts 148 and promote DSC autophagy and NK residence in decidua 21. Antiphospholipid antibodies-induced NLRP3-mediated IL-1β secretion in human trophoblasts could also be inhibited by rapamycin 204. In folate-deficient models, rapamycin alleviates impaired autophagy and defective decidualization 127, 128. However, rapamycin has also been reported for its potential in treating endometriosis 151 and endometrium-carcinoma 205 via enhancing NK cytotoxicity, reminding us that dose and side effects of rapamycin should be considered carefully when it comes to SM (**Table 3**).

To date, vitamin D has drawn great interests in reproductive system for its deficiency correlated with endocrine diseases and adverse pregnancy outcomes 206. Especially for pregnancy complications presented with poor placentation, decreased vitamin D could be a risk factor 207. A prospective cohort study conducted by Mumford *et al.* reports that sufficient preconception, instead of early pregnancy, concentrations of serum vitamin D are associated with better pregnancy outcomes among women who have suffered from one or two prior pregnancy losses before 208. Recent research indicates autophagy may participate in interactions between vitamin D and placental development, but whether it's mediated by vitamin D receptor (VDR) still remains controversial. Reduction of maternal serum vitamin D and intracellular VDR may lead to impaired vitality (decreased LC3B/Beclin-1) and reduced ratio of VTs in placenta 209. Additionally, the VDR protein is upregulated after supplementation without significant increase in its RNA and target gene level, indicating the importance of VDR but this effect is mediated by nongenomic response 210. However, according to Wilson *et al.*, though genetic deficiency of VDR caused aberrant autophagy with greater expression of Atg4b and activated mTOR, no statistically significant differences are observed in fetal growth, overall morphological parameters in VDR null placentae in comparison to controls 211. Vitamin D can rescue the impaired viability and invasion of trophoblasts induced by ROS-treated HTR-8 cell line 212. Fetal mortality, organ dysfunction and metabolic disorders could be alleviated by vitamin D supplementation in ischemia placenta via activating autophagy and attenuating apoptosis 210. During implantation and decidualization, vitamin D suppresses inflammation and positively regulates HOXA10 at maternal-fetal interface 213. Importantly, vitamin D decreases Th17 cells as well as the ratio of Th17 to Treg in peripheral blood of SM patients 214. Whether these effects are mediated by autophagy needs further confirmations. To be more specific, crosstalk among placenta, decidua and maternal serum vitamin D levels could play a critical role in pathogenesis of SM. Anyway, vitamin D should be a potential therapeutic method for SM in regulating immunological milieu and placentation (**Table 3**).

## Summary and future perspectives

Herein, we review the role of autophagy in regulating critical events during gestation, including fertilization and embryonic development, bio-functions of trophoblasts, placental development, decidualization, infiltration and residence of decidual NK, macrophage, and T cells. However, studies about autophagy in decidual macrophage and T cells are few, which needs more investigations. In addition, underlying mechanisms of RM mediated by aberrant autophagy are included, and thus we mention relevant potential therapeutic methods for SM by targeting autophagy. Particularly, rapamycin and vitamin D have been hotpots in recent decade for their multiple roles in regulating trophoblast survival, decidualization, or immunological milieu. More importantly, autophagy is a dynamic process and is involved in both maintaining homeostasis and causing pathological damage under stress. As a result, autophagy could act as a compensation in certain conditions which might lead to misinterpretations for results and mechanisms. Autophagy-related molecules might be new strategies for predicting SM, though the source of sampling and its power in scanning tests are still challenging problems. For example, Atg5 is not only a key factor in promoting trophoblast survival but also of great importance in regulating decidualization, monocyte-macrophage differentiation, mediating interaction between TGF-β and decidual T cells, possibly. Further well-designed studies are required to verify the role of autophagy in SM pathogenesis and the network of related regulatory mechanisms in order to make potential treatment methods into practice.

## Figures and Tables

**Table 1:** Expression of autophagy-related molecules on trophoblasts and decidua

Study	Objects	Samples	Expression of autophagy-related molecules
Avagliano, 2015	human	VTs	LC3, Bcl-2, Bax, HIF-1α
		EVTs	
Pan, 2018	human	VTs	LC3 and p62
Curtis, 2013	human	VTs	Beclin-1, Atg5, Atg9, Atg16L1, p62, LAMP-2
Roos, 2007	human	STBs	mTOR
Hung, 2017	human	term placentae	mTOR, TSC2, LC3B-II, p62
		CTBs	
Gauster, 2018	human	BeWo	ATGs, p62, LAMP-1, p53
		first trimester placentae	
Hung, 2010	human	CTBs	Bcl-2, Bcl-xL, LC3-II
Nakashima, 2020	human	STBs	TFEB, LAMP-1, LAMP-2, cathepsin D
		EVTs	
	mice	placentae	TFEB
Cao, 2016	human	PTB STBs	LC3, Atg16L1
		PTB CTBs	Atg7, Beclin-1
Hiyama, 2015	mice	STBs	LC3A, LC3B, LC3C
		giant trophoblast cells	LC3C
		spongiotrophoblast cells	LC3B
Zhu, 2019	mice	trophoblasts	LC3B-II, p62
Agrawal, 2015	mice	placentae	Atg4C, Atg7
Rhee, 2016	human	induced DSCs in vitro	LC3B
	mice	decidualizing cells	ACC, p-ACC, p-ULK1
Oestreich, 2020	human	induced DSCs in vitro	Atg16L, LC3B-II
	mice	decidualizing cells	
Su, 2020	human	decidua	LC3, p62
	mice		Atg5, LC3, cathepsin B, and p62
Lu, 2020	human	DSCs	LC3-II, p62
Oestreich, 2020	human	induced DSCs in vitro	FIP200

**Abbreviations**: ACC: acetyl-CoA carboxylase; Atg: autophagy-related genes; CTBs: cytotrophoblasts; DSCs: decidual stromal cells; VTs: villus trophoblasts; EVTs: extra-villus trophoblasts; FIP200: focal adhesion kinase family interacting protein of 200 kDa; HIF-1α: hypoxia-inducible factor-1α; LC3: microtubule-associated protein 1 light chain 3; mTOR: mammalian target of rapamycin; STBs: syncytiotrophoblasts; TSC2: tuberous sclerosis complex 2;PTB: preterm birth; ULK1: unc-51-like kinase 1; TFEB: transcription factor EB;

**Table 2:** Dysregulated autophagy and alterations of related molecules in RSM/SM

Study	Objects	Aberrant autophagy	Samples	Alterations of relevant molecules
Zhou, 2021	human	↑	villi in SM	increased Beclin1, LC3II/I, HMGB1
			LPS-treated HTR-8/SVneo	
Cai, 2018	human	↑	villi in SM	decreased MFN2; increased Beclin 1, LC3II/I, Atg5
Pan, 2021	human	↑	villi in RSM	decreased Shh, PTCH, SMO, Gli1/2/3
			JAR with Shh inhibition	increased LC3B, LC3II/I, LAMP1
Avagliano, 2015	human	↑	STBs/villi in SM	increased LC3, HIF-1α
			decidua	increased HIF-1α, Bax/Bcl-2, cleaved caspase 3
Jayaram, 2018	human	↑	PBMC in SM	decreased p62, hsp70
Yang, 2020	human	↓	villi in RSM	decreased PVT1
			HTR-8/SVneo with PVT downregulation	increased mTOR, p62; decreased Beclin-1, LC3- II/I, ULK1
Tan, 2020	human	↓	villi in RSM	decreased autophagosomes
Lu, 2020	human	↓	DSCs in SM	increased p62; decreased Atg5, LC3B, MITF, TNFRSF14, MMP9
Wei, 2020	rats	↓	placentae in a-PL rats	increased p62, mTOR; decreased Beclin-1, LC3-II

**Abbreviations**: aPL: antiphospholipid; Atg: autophagy-related genes; DSCs: decidual stromal cells; HIF-1α: hypoxia-inducible factor-1α; LC3: microtubule-associated protein 1 light chain 3; MMPs: matrix metalloproteinases; mTOR: mammalian target of rapamycin; PBMC: peripheral blood mononuclear cells; PVT1: plasmacytoma variant translocation 1; RSM: recurrent spontaneous miscarriage; Shh: Sonic Hedgehog signaling; SM: spontaneous miscarriage; STBs: syncytiotrophoblasts

**Table 3:** Potential treating methods for RSM/SM targeting autophagy

Targets	Supporting Study	Objects	Tissue/Cell line	Potential effects
PVT1	Yang, 2020	human	villi HTR-8/SVneo	• trigger autophagy via inactivating mTOR • facilitate invasion and adhesion ability of trophoblast cells
PLAC8	Feng, 2021	human	first trimester placentae decidua HTR-8 and JAR	• co-localize with p53 (an inhibitor of differentiation) and response for its degradation • upregulate AMPK, LC3II/I, Atg5, Atg12, and Beclin-1; downregulate p62 • promote trophoblastic viability and differentiation via enhancing autophagy • regulate trophoblast cell cycle and proliferation via p53 and its downstream molecule p21
Punicalagin	Wang, 2016	human	primary human trophoblasts	• activate autophagic flux in STBs • favor STBs survival via increased autophagy
Hyperoside	Wei, 2020	rats	placentae in vivo	• rescue pregnancy losses, exert effect of promoting autophagy and anti-inflammation • decrease IL-1β, IL-8, TF, ICAM-1, VCAM-1, complement C3, mTOR, p62 • increase LC3 and Beclin-1
Rapamycin	Wei, 2020	rats	placentae in vivo	• promote autophagy and suppress the inflammation • elevates LC3 stanning in junctional and labyrinth zone of placentae
	Mulla, 2018	human	EVTs	• suppress aPL-induced NLRP3-mediated IL-1β secretion
	Lu, 2020	human	decidua, dNK	• induce autophagy to reduce NK cytotoxicity • promote DSCs autophagy and mediate NK residence in decidua via MITF-TNFRSF14-MMP-adhesion molecules axis
		mice	placentae, uterus, dNK in vivo	
	Zhang, 2021	mice	induced DSCs in vitro in vivo	• rescue impaired decidualization in folate-deficiency conditions via activating autophagy by modulating AMPK/mTOR
	Chen, 2018	mice	DSC	• increase LC3II, Bax/Bcl-2 and reduce mTOR, p62 during decidualization
Vitamin D	Hutabarat, 2018	human	placentae	• corelate with MAP1LC3B/Beclin-1, involve in regulating trophoblast viability and placentation
	Tian, 2016	rats	placentae	• attenuate placenta apoptosis • upregulate Beclin-1 and LC3II/I placental trophoblast cells to reverse damage in PE model
	Pi, 2021	human	HTR-8	• promote trophoblastic viability and invasion • rescue impaired autophagy via increasing Beclin-1 and LC3-Ⅱ
	Rafiee, 2015	human	peripheral blood	• downregulate Th17/Treg ratio
Survivin	Pan, 2020	mice	embryo	• protect embryo from ROS-induced apoptosis and autophagy • regulate spindle organization and chromosome alignment

**Abbreviations:** AMPK: 5' adenosine monophosphate-activated protein kinase; aPL: antiphospholipid; Atg: autophagy-related genes; dNK cell: decidual natural killer cell; DSCs: decidual stromal cells; IL: interleukin; LC3: microtubule-associated protein 1 light chain 3; MMPs: matrix metalloproteinases; mTOR: mammalian target of rapamycin; PE: preeclampsia; RSM: recurrent spontaneous miscarriage; SM: spontaneous miscarriage; STBs: syncytiotrophoblasts; Th: T helper cell; Treg: regulatory T cells

## Abbreviations

AMPK: 5' adenosine monophosphate-activated protein kinase
Atg: autophagy-related genes
ATP: adenosine triphosphate
CTBs: cytotrophoblasts
CXCL12: C-X-C motif chemokine ligand 12
CXCR4: C-X-C motif chemokine receptor 4
dNK cell: decidual natural killer cell
DSCs: decidual stromal cells
ER stress: endoplasmic reticulum stress
ESCs: endometrial stromal cells
EVTs: extra-villus trophoblasts
E2: estradiol
FGR: fetal growth restriction
FIP200: focal adhesion kinase family interacting protein of 200 kDa
FOXO1: forkhead box O1
HF/HS: high-fat/high-sugar
HIF-1α: hypoxia-inducible factor-1α
HOXA10: homeobox A10
IDO: indoleamine 2,3-dioxygenase
IGF2: Insulin-like growth factor 2
IL: interleukin
LC3: microtubule-associated protein 1 light chain 3
LncRNA: long non-coding RNA
MMPs: matrix metalloproteinases
miR: micro-RNA
mTOR: mammalian target of rapamycin
mTORC1: mTOR complex 1
NF-κB: nuclear factor kappa-B
NK cell: natural killer cell
PE: preeclampsia
PI3K: class I phosphatidylinositol 3-kinase
PLAC8: Placenta-specific protein 8
PVT1: plasmacytoma variant translocation 1
P4: progesterone
ROS: reactive oxygen species
RSM: recurrent spontaneous miscarriage
Shh: Sonic Hedgehog signaling
SM: spontaneous miscarriage
STBs: syncytiotrophoblasts
TFEB: transcription factor EB
TGF-β: Transforming growth factor beta
Th: T helper cell
TNF-α: Tumor Necrosis Factor alpha
Treg: regulatory T cells
ULK1: unc-51-like kinase 1
uNK cell: uterine NK cell
VDR: vitamin D receptor
VEGF: vascular endothelial growth factor
VEGFA: vascular endothelial growth factor A
VTs: villus trophoblasts
3-MA: 3-methyladenine
